# Value of cerebrovascular hemodynamics and thromboelastogram-related parameters in evaluating the effect of Naomai Jiejing Decoction on the rehabilitation of patients with aneurysmal subarachnoid hemorrhage

**DOI:** 10.4314/ahs.v25i1.21

**Published:** 2025-03

**Authors:** Xuetao Wang, Zesen Lin, Dan Jin, Rui Huang, Tao Kang, Wenjun Su

**Affiliations:** Neurosurgery, Zhongshan Hospital of Traditional Chinese Medicine, Zhongshan, China

**Keywords:** Aneurysmal subarachnoid hemorrhage, cerebrovascular hemodynamics, thromboelastogram, Naomai Jiejing Decoction, rehabilitation outcome, clinical efficacy

## Abstract

**Background:**

To explore the value of cerebrovascular hemodynamic and thromboelastogram-related parameters in evaluating the effect of Naomai Jiejing Decoction (NJD) on the rehabilitation of patients with Aneurysmal subarachnoid hemorrhage (aSAH).

**Methodology:**

This article is observational studies. This study compared cerebrovascular hemodynamics, thromboelastogram results, and rehabilitation outcomes between a research group (treated with Naomai Jiejing Decoction) and a control group (not treated with Naomai Jiejing Decoction) of 160 patients with Aneurysmal subarachnoid hemorrhage. Logistic regression analysis explored associations between poor rehabilitation outcomes and hemodynamic/thromboelastogram-related parameters in the research group.

**Results:**

At 12 months after operation, the measured hemodynamics values were lower in research group than those in control group, and the differences were statistically significant (P<0.05). Patients with good rehabilitation outcomes had lower measured hemodynamics values than those with poor rehabilitation outcomes. The results of Logistic regression analysis showed that the high values in hemodynamics and thromboelastogram examinations were risk factors for poor rehabilitation outcomes in research group (P<0.05).

**Conclusion:**

Monitoring cerebrovascular hemodynamic and thromboelastogram-related parameters in patients with Aneurysmal subarachnoid hemorrhage receiving Naomai Jiejing Decoction treatment can offer valuable prognostic and therapeutic insights, with improvements correlating to better rehabilitation outcomes, underscoring their significance in assessing intervention effectiveness.

## Introduction

Aneurysmal subarachnoid hemorrhage (aSAH) is a common clinical syndrome in which blood directly enters the sub-arachnoid space due to aneurysm rupture and bleeding. Its common clinical manifestations are headache, fever, arrhythmia, and even cerebral hernia and even death in severe cases^1^. Therefore, it remains an important topic in clinical research to explore an efficient method for clinical examination, diagnosis and prognostic prediction of aSAH, which is of great significance for reducing the mortality and disability rate. With the deepening of related research, cerebral vasospasm (continuous reduction of vessel diameter) and changes in the coagulation state (hypercoagulability) have often been detected in aSAH patients^2^. Characterized by non-invasiveness and repeatability, cerebrovascular hemodynamics can intuitively and clearly reflect the cerebral blood flow status of the subject, and help doctors effectively evaluate and analyze the conditions of cerebral ischemia, hemorrhage and vasoconstriction, and relaxation of patients^3^. Thromboelastogram (TEG) is an important indicator for the dynamic changes in blood coagulation, which can accurately detect the activity of coagulation factors and the level of fibrinogen, and fully reflect the changes in the body's coagulation state, making it an auxiliary reference for the severity assessment of aSAH^4^. At present, there are few reports on the clinical association between cerebrovascular hemodynamic and TEG-related parameters and postaSAH rehabilitation outcomes in China. This study aims to compare the differences in hemodynamics, TEG parameters, and rehabilitation outcomes between aSAH patients who receive Naomai Jiejing Decoction (NJD) and those who do not, in order to investigate the utility of hemodynamics and TEG in evaluating the efficacy of NJD. The objective is to provide insights for clinical diagnosis and prediction methods in the treatment of aSAH with NJD.

## Patients and methods

### Patients

Totally 160 aSAH patients admitted to Zhongshan Traditional Chinese Medicine Hospital from January 2020 to December 2022 were selected as the subjects, and divided into research group (80 cases, treated with NJD) and control group (80 cases, not treated with NJD). All patients underwent cerebrovascular hemodynamics and TEG examinations. There were no statistically significant differences in age, sex, diameter of aneurysm, hunt-Hess grade at admission, preoperative Hunt-Hess grad, intraoperative aneurysm rupture and perioperative complicationsbetween the two groups (P>0.05) (Table 1). The subjects voluntarily signed the informed consent form, and this study was approved by the Medical Ethics Committee.

**Table 1:** Comparison of general data between the two groups (χ̅ ± s, n)

Clinical data		Research Group (n=80)	Control Group (n=80)	x^2^/t	P
Gender [n (%)]	Male	44 (55.00)	47 (58.75)	0.287	0.592
	Female	36 (45.00)	33 (41.25)		
Mean age (Y)		45.84±6.12	46.02±6.38	0.182	0.856
Mean diameter of aneurysm (cm)		0.66±0.12	0.69±0.15	1.397	0.164
	I-II	36 (45.00)	37 (46.25)		
Hunt-Hess grade at admission [n (%)]	III-IV	43 (53.75)	40 (50.00)	1.403	0.496
	V	1 (1.25)	3 (3.75)		
	I-II	34 (42.50)	39 (48.75)		
Preoperative Hunt-Hess grade [n (%)]	III-IV	46 (57.50)	38 (47.50)	5.131	0.077
	V	0 (0)	3 (3.75)		
Intraoperative aneurysm rupture [n (%)]	Yes	22 (27.50)	24 (30.00)	0.153	0.696
	No	58 (72.50)	56 (70.00)		
Perioperative complications [n (%)]	Yes	19 (23.75)	21 (26.25)	0.767	0.683
	No	61 (76.25)	59 (73.75)		

### Screening criteria for aSAH

All patients were diagnosed with aneurysms by digital subtraction angiography and/or CT angiography and with SAH by CT. All cases were confirmed by surgery5. All patients were diagnosed with aSAH based on characteristic findings on CT or digital subtraction angiography, including the presence of blood in the subarachnoid space and evidence of aneurysm rupture.

### Inclusion and exclusion criteria

Inclusion criteria: 1) Patients aged ≥20 years and ≤60 years, and 2) those with complete baseline data. Exclusion criteria: 1) Pregnant and lactating women, 2) patients with a history of underlying cerebrovascular diseases or severe systemic underlying diseases, 3) those with serious coagulation disorders, 4) those with intracranial tumors, 5) those with fatal systemic diseases, 6) those who were hospitalized for fewer than 3 days, 7) those who or whose families refused the treatment protocol, or 8) those with severe neurological dysfunction caused by intracranial hematoma during hospitalization.

## Methods

### Therapeutic methods

In research group, preoperative basic treatment + nimodipine + NJD No.1 were given. As a granular formulation, NJD No.1 consisted of 30 g each of Cornu bubali (decocted first), Polygonum cuspidatum, Radix Paeoniae Alba and Fructus Chaenomelis, 15 g of Radix Puerariae, 9 g of Gentina scabra Bunge, and 1.5 g of artificial bovine calculus powder (administered after dissolved), one dose per day. Besides, postoperative basic treatment + nimodipine (Jiangsu Kanion Pharmaceutical Co., Ltd., NMPN H20066423, specification: 60 mg, taken orally, 60 mg/time, bid) + NJD No.2 (NJD No.1, 10 g each of Semen Pruni Persicae and Carthamus tinctorius L, 3 g of Hirudo, granular formulation, one dose per day) were given. In control group, basic treatment + nimodipine (the treatment mode was the same as that in research group) were given. Cerebrovascular hemodynamics and TEG examinations were performed in both groups, and all patients were followed up for one year. Rehabilitation outcomes were assessed using the Glasgow outcome scale (GOS). GOS scores were given by outpatient reexamination and telephone inquiry, and only postoperative GOS scores were recorded for dead patients and those who voluntarily quit the study. According to the GOS scores, the patients in research group were divided into good group (GOS score ≥3 points) and poor group (GOS score <3 points).

### Examination methods

Cerebrovascular hemodynamics: The extracranial internal carotid artery (ICA) and blood vessels near the Willis ring were routinely detected using an EMS-9PB hemodynamic analyzer (DELICA, China) (spectrum scale: cm/s). The mean values of peak systolic velocity (Vs), mean velocity (Vm), peak diastolic velocity (Vd), pulsation index (PI), and resistance index (RI) were analyzed and processed by computer. Hemodynamics examinations were performed at 2 d after operation (before treatment with NJD in research group) and 12 months after operation (after treatment with NJD in research group). TEG: The TEG examination refers to Thromboelastography, which is a laboratory test used to assess a patient's overall hemostatic function. It provides information about the clot formation and breakdown processes, giving a comprehensive picture of the patient's coagulation status. During a TEG test, a small sample of the patient's blood is placed in a specialized cup or cuvette that contains activators to initiate clotting. The sample is then subjected to controlled oscillation, and changes in the viscoelastic properties of the clot are measured and recorded in real-time. TEG examination was conducted at 2 d and 12 months after operation using a DRNX-III TEG instrument. The platelet aggregation function was evaluated by maximum amplitude (MA, normal reference range: 50-70 mm). MA<45 mm was considered as platelet hypofunction, and MA>70 mm as platelet hyperfunction. The coagulation index (CI, normal reference range: -3-3) was calculated. CI<-3 was considered as hypocoagulation, and CI>3 as hypercoagulation.

### Statistical analysis

Statistical Product and Service Solutions (SPSS) 25.0 software (IBM, Armonk, NY, USA) was used for data processing. Enumeration data were described by percentage (%) and compared by x2 test between two groups. Measurement data of normal distribution were described by (χ̅ ± s) and compared by independent-samples t-test between two groups and by paired t-test within the group. The associations between cerebrovascular hemodynamics and TEG-related parameters and poor rehabilitation outcomes in research group were explored by Logistic regression analysis. P<0.05 was considered statistically significant.

## Results

### Comparison results of hemodynamic parameters between the two groups

There were no statistically significant differences in the measured values of Vs, Vm, Vd, PI, and RI between the two groups at 2 d after operation (P>0.05). At 12 months after operation, the measured values of Vs, Vm, Vd, PI, and RI were lower in research group than those in control group, and the differences were statistically significant (P<0.05) (Table 2 and 3).

**Table 2:** Comparison of measured values of Vs, Vm and Vd between the two groups (χ̅ ± s, cm/s)

Group	Case	Vs		Vm		Vd	

		2 d after operation	12 months after operation	2 d after operation	12 months after operation	2 d after operation	12 months after operation
Research Group	80	138.78±26.45	98.78±22.54	80.54±13.54	60.11±8.46	53.02±7.98	43.84±5.49
Control Group	80	137.45±25.97	107.84±23.12	80.97±14.03	68.84±9.97	53.54±8.12	46.87±5.88
t	-	0.321	2.510	0.197	5.972	0.409	3.369
P	-	0.749	0.013	0.844	<0.001	0.683	0.001

**Table 3:** Comparison of measured values of PI and RI between the two groups (χ̅ ± s)

Group	Case	PI		RI	

		2 d after operation	12 months after operation	2 d after operation	12 months after operation
Research Group	80	1.22±0.12	0.67±0.08	1.66±0.35	1.11±0.12
Control Group	80	1.24±0.13	0.83±0.11	1.64±0.34	1.32±0.15
t	-	1.011	10.522	0.367	9.778
P	-	0.314	<0.001	0.714	<0.001

### Comparison results of TEG findings between the two groups

There were no statistically significant differences in the measured values of MA and CI between the two groups at 2 d after operation (P>0.05). At 12 months after operation, the measured values of MA and CI were lower in research group than those in control group, and the differences were statistically significant (P<0.05) (Table 4).

**Table 4:** Comparison results of TEG findings between the two groups (χ̅ ± s)

Group	Case	MA (mm)		CI	

		2 d after operation	12 months after operation	2 d after operation	12 months after operation
Research Group	80	76.84±5.58	63.15±3.15	3.95±0.64	2.11±0.41
Control Group	80	77.03±6.01	69.88±4.35	3.91±0.61	2.76±0.53
t	-	0.207	11.208	0.405	8.676
P	-	0.836	<0.001	0.686	<0.001

### Associations between cerebrovascular hemodynamic and TEG-related parameters and poor rehabilitation outcomes in research group

Good group had lower measured values of Vs, Vm, Vd, PI, RI, MA and CI than poor group, showing statistically significant differences (P<0.05) (Table 5). Logistic regression analysis was performed with hemodynamic and TEG-related parameters as independent variables and rehabilitation outcomes in research group as dependent variables (GOS score ≥3 points: 0, GOS score <3 points: 1). The results showed that the high values of Vs, Vm, Vd, PI, RI, MA, and CI in hemodynamics and TEG examinations were risk factors for poor rehabilitation outcomes in research group (P<0.05) (Table 6).

**Table 5:** Comparison results of hemodynamics and TEG findings among patients with different rehabilitation outcomes in research group (χ̅ ± s)

Group	Case	Vs (cm/s)	Vm (cm/s)	Vd (cm/s)	PI	RI	MA (mm)	CI
Good Group	63	95.52±21.86	57.14±7.96	42.41±5.31	0.62±0.07	1.03±0.09	61.20±2.66	1.77±0.34
Poor Group	17	110.87±25.06	71.13±10.33	49.13±6.14	0.85±0.10	1.39±0.24	70.38±4.97	3.38±0.68
t	-	2.490	6.022	4.478	10.913	9.749	10.273	13.632
P	-	<0.001	<0.001	<0.001	<0.001	<0.001	<0.001	<0.001

**Table 6:** Logistic regression analysis of cerebrovascular hemodynamic and TEG-related parameters in poor rehabilitation outcomes in research group

Group	*β*	*SE*	*Wald χ^2^*	P	*OR value*	95% CI
Vs	1.186	0.397	8.923	<0.001	3.274	1.108-5.440
Vm	1.267	0.388	10.662	<0.001	3.550	1.106-5.994
Vd	1.234	0.393	9.856	<0.001	3.435	1.113-5.757
PI	1.523	0.372	16.761	<0.001	4.595	1.185-8.005
RI	1.467	0.386	14.442	<0.001	4.336	1.176-7.496
MA	1.519	0.379	16.060	<0.001	4.568	1.191-7.945
CI	1.677	0.392	18.302	<0.001	5.349	1.167-9.531

## Discussion

aSAH is a common hemorrhagic cerebrovascular disease in neurosurgery^6^. Under a normal state, an arachnoid membrane is a translucent membrane primarily composed of connective tissues and filled with cerebrospinal fluid, which mainly exerts a protective effect on brain tissues^7^. After the onset of aSAH, a large amount of blood will flow into the subarachnoid space, causing serious damage to the brain and inducing headache, the elevation of intracranial pressure, and other symptoms. In TCM diagnosis and treatment, aSAH mostly belongs to the categories of “headache due to blood stasis” and “stroke”. Dysfunction of qi and blood and blood stagnation in viscera resulting from internal deficiency, endogenous damp phlegm, and yang transformation into endogenous wind are considered the major causes of aSAH and headache symptoms. In the prescription of NJD, Cornu bubali as a monarch drug has effects of clearing away heat and removing toxicity, dispersing blood stasis and relieving pain. Radix Paeoniae Alba and Fructus Chaenomelis as ministerial drugs have effects of nourishing blood for regulating menstruation, nourishing the liver and relieving pain, relaxing muscles and activating collaterals. Radix Puerariae and Gentina scabra Bunge as adjuvant drugs can invigorate yang and relieve diarrhea, induce the discharge of measles toxicity, clear heat, and purge fire. Artificial bovine calculus powder as envoy drugs can eliminate phlegm and arrest convulsion. With the combination of these drugs, NJD can promote blood circulation and remove blood stasis, dredge collaterals, and relieve pain, thereby achieving significant therapeutic effects on headache and the elevation of intracranial pressure.

In actual diagnosis and treatment, aSAH is treated as an acute and critical illness with high mortality and permanent disability rates. It is of great significance to explore an efficient method for the early diagnosis and prognostic prediction of aSAH for reducing mortality and disability rates. In this study, therefore, the application value of hemodynamics and TEG in the treatment of aSAH with NJD was deeply studied. The results showed that the measured values of Vs, Vm, Vd, PI, and RI were lower in research group at 12 months after operation and in patients with good rehabilitation outcomes. It was found by Logistic regression analysis that the high values of Vs, Vm, Vd, PI and RI were risk factors for poor rehabilitation outcomes in research group (P<0.05), suggesting that the changes in levels of cerebrovascular hemodynamics-related indicators may be related to the changes in the condition of aSAH patients.

The reason is as follows: As mentioned above, a large amount of blood will flow into the subarachnoid space following aneurysm rupture, causing SAH. Due to hemorrhage symptoms, intracranial pressure may be increased, so blood produces mechanical stimulation against the vascular wall and destroys the structure of vascular wall, worsening endothelial cell injury. As a result, a large number of oxygen-free radicals and inflammatory factors are released, triggering inflammatory and stress responses, enhancing vasoconstriction, and forming a vicious circle. Eventually, brain tissues are seriously damaged^8^,^9^.

Moreover, the destruction and dissolution of red blood cells due to hemorrhage symptoms may facilitate the synthesis and release of vasoconstrictors such as endothelin, so that strong cerebrovascular contraction and vasospasm are caused and the risk of delayed cerebral ischemia is increased^10^. In hemodynamics, Vs, Vm, and Vd can directly and clearly reflect the cerebral blood supply status of subjects,nd their levels are closely related to the changes in intracranial pressure. PI and RI calculated based on Vs, Vm, and Vd can effectively reflect the cerebrovascular resistance.

The high levels of PI and RI indirectly suggest that the subject develops significant cerebral blood flow blockage and may have an elevation of intracranial pressure and lumen stenosis. There is a study showing that the cerebral artery has an autoregulation function, so cerebral blood supply status in patients with cerebrovascular diseases is often evaluated and analyzed by dynamic observation and continuous monitoring^11^. The risk of post-aSAH cerebral vasospasm is generally high, and cerebral resistance vessels affected by the cerebrovascular autoregulation function reactively expand, hemodynamically manifested as high flow velocity. At the same time, under the influence of increased intracranial pressure, the cerebrovascular resistance is enhanced, and accordingly, the measured values of Vs, Vm, Vd, PI, and RI in aSAH patients significantly rise. In this study, the hemodynamics-related indicators were better improved in research group after treatment with NJD, indicating that NJD can achieve ideal therapeutic effects. To sum up, in the cerebrovascular hemodynamics examination for aSAH patients after treatment with NJD, significant decreases in the levels of Vs, Vm, Vd, PI, and RI can indirectly demonstrate that intracranial hypertension and cerebral vasospasm are greatly ameliorated, and the condition of patients may be improved.

The results of the TEG examination showed that the measured values of MA and CI were lower in research group at 12 months after operation and in patients with good rehabilitation outcomes. It was found by Logistic regression analysis that the high values of MA and CI were risk factors for poor rehabilitation outcomes in research group (P<0.05), suggesting that the changes in the levels of TEG-related parameters may be related to the changes in the condition of aSAH patients. The reason is as follows: As mentioned above, aSAH can cause elevation of intracranial pressure and vasospasm, worsen vascular wall and endothelial cell injury, and contribute to the release of oxygen free radicals, inflammatory factors, and vasoconstrictors, thus aggravating the inflammatory and stress responses. Under the regulation of the immune system, in order to control the development of disease, platelets can quickly aggregate at sites of bleeding and injury to form a thrombus, thus achieving hemostasis and coagulation. In actual diagnosis and treatment, however, aSAH is treated as an acute and critical illness prone to multiple and sudden variabilities, which cause serious damage to brain tissues, and may lead to continuous activation of coagulation system, thus increasing the risk of thrombosis and delayed cerebral ischemia. Moreover, aSAH may cause excessive activation of platelets and then result in platelet inertia, affecting the physiological function of platelets and ultimately seriously affecting the function of the coagulation-fibrinolysis system^12^,^13^. By observing and recording the dynamic changes in blood viscosity during coagulation and depicting the coagulation-fibrinolysis process in a graphic form, TEG examination can help doctors intuitively and clearly observe the coagulation state of patients^14^,^15^. In this study, the measured values of MA and CI in research group after treatment with NJD were lower and basically within the normal referenceanges, suggesting that NJD has a positive effect on improving the coagulation-fibrinolysis system of aSAH patients. To sum up, in the TEG examination for aSAH patients after treatment with NJD, decreases in the levels of MA and CI can indirectly demonstrate that platelet overactivation is relieved to some extent, and the risk of thrombosis and cerebral vasospasm declines, thereby ameliorating intracranial pressure and the condition of disease. Meanwhile, with the support of modern medicine, hemodynamics examination has the advantages of non-invasiveness and repeatability, and TEG examination has the virtue of short detection duration, many references and simple operation. Therefore, hemodynamics combined with TEG can effectively reflect the cerebrovascular blood flow, resistance, and coagulation state of patients in the efficacy evaluation, and help doctors evaluate and analyze the condition of patients more accurately and comprehensively. This study had deficiencies as follows: 1) The sample size was limited. 2) The duration from onset to admission, surgical modes, and pathological types may have a certain influence on the condition and prognosis of aSAH patients, but the patients were not further grouped for comparison, and the broad effectiveness of cerebrovascular hemodynamics and TEG-related parameters in the clinical evaluation of aSAH was not verified.

## Conclusion

Cerebrovascular hemodynamic and TEG-related parameters are at high levels in poor rehabilitation outcomes after treatment of aSAH with NJD. During the treatment of aSAH patients with NJD, a decline in cerebrovascular hemodynamic and TEG-related parameters can indirectly suggest that the condition of patients may be improved and significant therapeutic effects are achieved.
